# Clearance and persistence of *Escherichia coli* in the freshwater mussel *Unio mancus*

**DOI:** 10.1038/s41598-022-16491-x

**Published:** 2022-07-20

**Authors:** M. Campos, L. Lobato-Bailón, R. Merciai, O. Cabezón, I. Torres-Blas, R. Araujo, L. Migura-Garcia

**Affiliations:** 1 Wildlife Conservation Medicine Research Group (WildCoM), Departament de Medicina i Cirurgia Animals, Universitat Autònoma de Barcelona, 08193 Bellaterra, Spain; 2 Freshwater Mussel Breeding Laboratory of Lake Banyoles, Consorci de l’Estany, Plaça dels Estudis 2, 17820 Banyoles, Spain; 3 Research and Conservation Department, Zoo de Barcelona, Parc de la Ciutadella s/n, 08003 Barcelona, Spain; 4 UAB, Centre de Recerca en Sanitat Animal (CReSA, IRTA-UAB), Campus de la Universitat Autònoma de Barcelona, 08193 Bellaterra, Spain; 5 MNCN, Museo Nacional de Ciencias Naturales-CSIC, C/José Gutiérrez Abascal 2, 28006 Madrid, Spain; 6 Unitat mixta d’Investigació IRTA-UAB en Sanitat Animal, Centre de Recerca en Sanitat Animal (CReSA), Campus de la Universitat Autònoma de Barcelona (UAB), 08193 Bellaterra, Spain; 7 IRTA, Programa de Sanitat Animal, Centre de Recerca en Sanitat Animal (CReSA), Campus de la Universitat Autònoma de Barcelona (UAB), 08193 Bellaterra, Spain

**Keywords:** Biological techniques, Ecology, Microbiology, Zoology, Environmental sciences

## Abstract

The excessive use of antibiotics has led to the emergence of resistant bacteria, mainly from the Enterobacterales group, with high pathogenic/zoonotic potentials that can lead to problems in public health. The increasing presence in freshwater ecosystems highlights the need to evaluate potential sentinel species as risk indicators for both ecosystem and human health. The freshwater mussels provide several ecosystem services, may represent potential sentinel species due to their ability to filter water and retain both organic and inorganic particles. We tested the capability of *U. mancus* to retain *Escherichia coli* as a model bacterial organism. Under experimental conditions, the mussels could clear suspended *E. coli*, facilitating its rapid elimination from water within the first 24 h after exposure. The species also presented a maximum retention time of 4 days. We also provide allometric equations correlating the filtering capacity with the length and the weight of mussel body parts often used in biometric studies. We provide a first assessment of the potential of the bivalve *Unio mancus* to act as a sentinel species for the detection of Enterobacterales and demonstrate the ability to act as a water cleaner.

## Introduction

One Health is a collaborative, multisectoral, and transdisciplinary approach with the goal of achieving optimal health outcomes recognizing the interconnection between humans, animals, plants, and their shared environment. Although the role of the environment is still scarcely acknowledged in public health research, it is well known that the integration of environmental factors into the One Health strategy leads to a holistic understanding of planetary health. Studying the ecology of pathogens in the environment, in particular, can help to identify health risks, potentially preventing widespread exposure to such threats.

An extensive literature illustrates the link between anthropogenic activity and the occurrence of several important public health issues, such as emerging infectious diseases (EID) and antibiotic resistance^1,2^. Indeed, the overuse of antibiotics has led to the appearance of resistant bacteria that pose a global health risk. For example, the Enterobacterales are considered zoonotic and have pathogenic potential^3–5^. Considering that more than 70% of EIDs are zoonotic and most of them originate from wild species^6–8^, the potential role that wild animals can play as sentinels in the detection and surveillance of zoonotic pathogens has been extensively recognized^9–11^.

Inland waters, such as rivers, lakes and swamps, are the hotspots of zoonotic agents originating from human activities^12,13^. Despite this, the importance of aquatic ecosystems in the dispersal and mitigation of pathogens is still not fully understood. Knowledge of the factors facilitating the emergence and dispersion of potential pathogens is necessary to establish appropriate public health measures that can alleviate future threats.

Freshwater mussels provide a valuable ecosystem service to the aquatic environment by filtrating a large quantity of water during the feeding process. These sedentary organisms dwell in the sediments of water bodies and consume bacteria, phytoplankton, detritus and organic matter, among others. Through these feeding habits, the mussels translocate nutrients along the water column, providing resources to other organisms and helping to maintain the quality of the aquatic environment^12,14–17^.

Most of the literature on freshwater mussels acting as sentinels has focused on the bioaccumulation of contaminants and pathogens by a limited number of species, including the invasive zebra mussel *Dreissena polymorpha*^18^, the Asian clam *Corbicula fluminea*^19^ and the Alabama rainbow mussel *Villosa nebulosa*, a native North American naiad^17^. Several studies investigating the bioaccumulation and elimination kinetics of microorganisms by bivalves have determined their carrying capacity regarding bacterial, viral and protozoan pathogens, such as *Escherichia coli* and *Clostridium perfringens*^20^, the avian influenza virus^21^, the Norwalk-like virus^22^ and *Toxoplasma gondii*^23,24^, among others. *Dreissena polymorpha*, in particular, is considered to have a high potential to reduce *E. coli* counts in freshwater systems^25^. Moreover, bivalves have the ability to filter and accumulate pharmaceuticals and other chemical compounds from aquatic ecosystems, making these animals ideal biological indicators for ecotoxicological studies^12,26–28^.

*Unio mancus* (Phylum Mollusca; Class Bivalvia) is a freshwater mussel, or naiad, inhabiting inland Mediterranean freshwaters in northeastern Spain, France and Italy. It is classified as near threatened in the *International Union for Conservation of Nature* (*IUCN*) Red List^29^ and as vulnerable in the Spanish National Catalogue of Endangered Species. The species has a fragile life cycle and needs a native host fish for its larvae development and dispersal^30,31^. Its populations have been in decline mainly due to habitat loss, pollution and invasive species introductions that disrupt their life cycle.

*Unio mancus* plays an important role in preserving and restoring water quality in impaired Mediterranean ecosystems^32,33^. However, its potential to act as a sentinel species has not yet been demonstrated^28,34^. The objectives of the present study were to determine the capacity of *U. mancus* to clear *E. coli* from water, to observe the viability of this bacterium in the soft tissue of the mussels, and to calculate the retention time under experimental conditions, in order to assess the mussel’s potential to act as a sentinel species in anthropogenically altered freshwater environments in the Mediterranean rivers.

## Results

### Mussel biometry

Necropsies were performed on the sacrificed specimens to study some biometric characteristics of *U. mancus*, including length and soft body and shell weights (Table 1). A total of 38 3-year-old specimens with an average length of 25.69 ± 3.99 mm (µ ± SD) was analysed. The average specimen weight (soft body, shell and free water) was 2.27 ± 0.95 g. The average soft body (wet weight) and shell weight was 0.52 ± 0.24 g and 1.07 ± 0.49 g, respectively. The wet mass of interbranchial liquids was calculated for all samples by subtracting the weight of the different parts from the total weight. The shell, soft body and free water, respectively, accounted for 46.4%, 22.8% and 30.8% of the total weight. The mean condition index value, calculated from the length and the weight of the soft body, was 59.5 ± 27.8 mm/g. Interestingly, variability between the mean and the standard deviation of the condition index was estimated to be 46.7%.

**Table 1:** Biometric parameters and linear relationships.

*N* = 38	Mean	Min	Max	% of total W
L (mm)	25.7 ± 4.0	17.51	33.61	
Total W (g)	2.3 ± 0.9	0.6	4.1	
Shell W (g)	1.1 ± 0.5	0.3	2.0	46.4 ± 5.3
Soft body W (g)	0.5 ± 0.2	0.1	1.1	22.8 ± 4.8
Free water W (g)	0.7 ± 0.3	0.2	1.3	30.8 ± 7.0
Condition index (L/soft body W)	59.5 ± 27.8	26.7	175.1	46.7*

Relationship	*R*^2^	Slope (a)	Intercept (b)	
Total W with L	0.88	0.2218	− 3.4267	
Shell W with L	0.81	0.1099	− 1.7523	
Soft body W with L	0.79	0.054	− 0.8655	
Free water W with L	0.62	0.0579	− 0.8089	
Dry soft body W (estimated) with L	0.79	0.0092	− 0.1471	

The biometric parameters of *U. mancus* specimens: Shell length (L, mm) and weight (W, g) of the different parts of the mussel are shown. Linear relationships (W = aL + b) and correlation coefficients (*R*^2^) are also shown. The dry soft body weight was estimated considering a soft tissue water content of 83%, according to Mo and Neilson^35^. The percentage of variation of the condition index was calculated between the mean and the standard deviation (*). *N* sample size, *Min.* minimum, *Max.* maximum.

**Figure 1:**
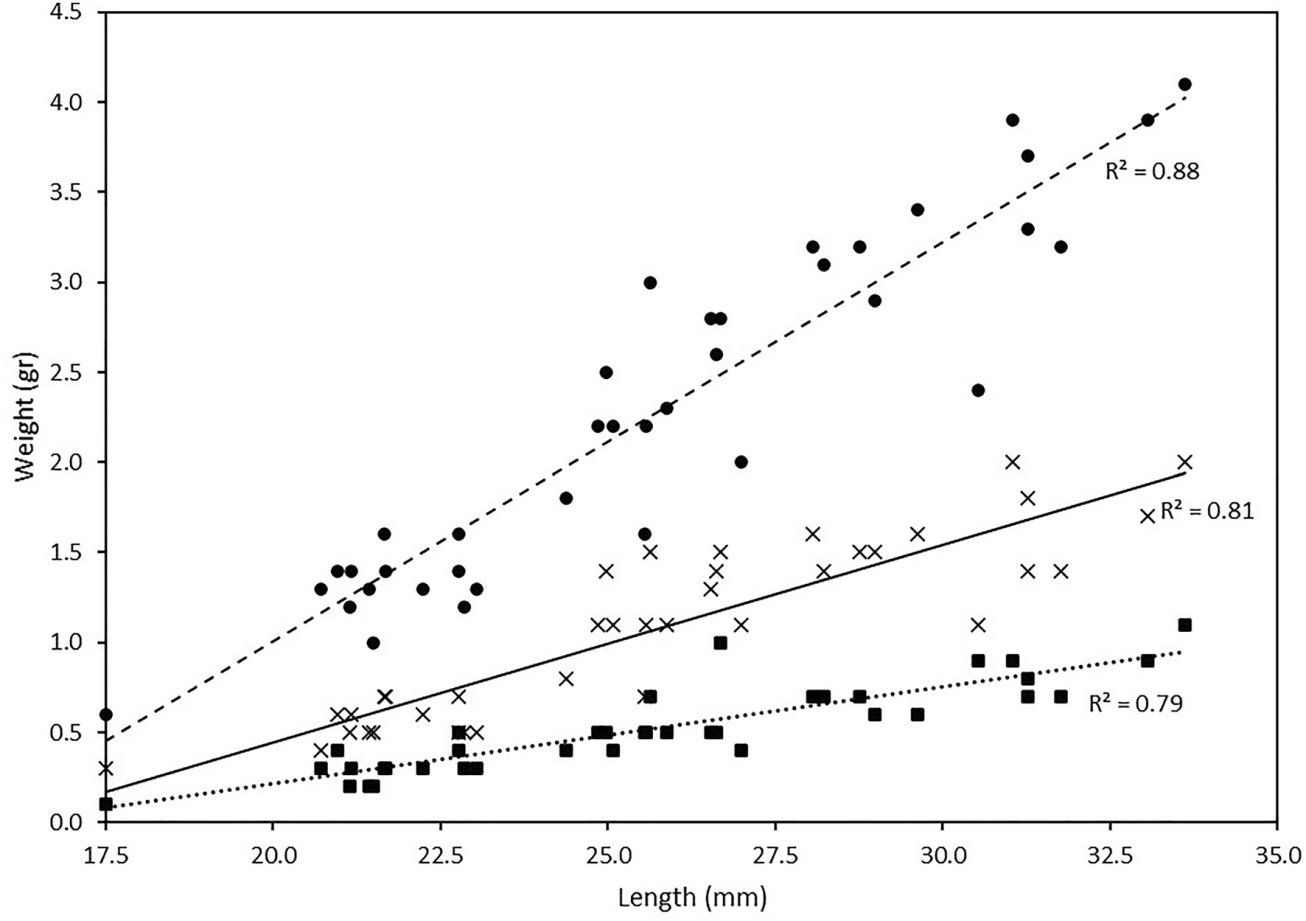
Relationship between *U. mancus* length shell (mm) and weight (g) [total weight (circle), soft body weight (times) and shell weight (square)]. The linear correlation and the coefficient of determination (*R*^2^) are indicated for each relationship.

The relationship between total weight of the different body parts (both with and without the shell) and length was calculated for each specimen (Fig. 1). Except for free water parameter, the R^2^ values ranged from 0.79 to 0.88, indicating a good model fit. The relationship between free water weight and length presented the smallest R^2^ value (0.62). This parameter represents the interbranchial or internal cavity liquid weight, which is subject to greater estimation error and, consequently, greater variability. The soft tissue was used for the bacteriological analysis and, therefore, its dry weight, which is often used in filtration ratio studies, could not be directly measured. However, an approximation of dry weight was obtained by considering the water content of mussel soft tissue is around 83%^35^.

### Mussel bacterial clearance

The filtering capacity of the mussels was assessed from nine water samples collected after the exposure phase (Table 2). The average elimination value of *E. coli* was 6.8 × 10^4^ ± 1.6 × 10^4^ CFU/mL, corresponding to 45.1 ± 10.7% of the inoculated bacteria. Clearance and filtration rates were also assessed. Clearance rate refers to a volume of water cleared of suspended particles, while filtration rate is a pumping or volume flow rate^36^. A clearance rate of 18 ± 4 mL/h was calculated for *U. mancus* on the basis of bacterial elimination. To estimate the filtration rate, we used an equation previously established for unionids at 20 °C based on the soft body weight of *U. terminalis* [F = 116 W^0.605^, F = filtration (mL/h), W = soft body wet mass weight (g)]^37^. The estimated filtration rate was 76.0 mL/h. However, no significant correlation was found between shell length and observed filtering capacity (Clearance rate: Spearman's *ρ* = 0.144, P value = 0.711; Filtration rate: Spearman's *ρ* = − 0.119, P value = 0.761). Thus, the correlation between soft body wet mass weight and estimated filtration ratio was significant, as expected, since it was calculated with the weight of the mass of the soft tissues (Spearman's *ρ* = 0.822, P value = 0.007**).

**Table 2:** Filtration analysis.

N = 9	Average	Min	Max
L (mm)	24.2 ± 2.1	20.9	26.68
Total W (g)	2.1 ± 0.6	1.4	3
Soft body W (g)	0.51 ± 0.22	0.3	1
Average CFU/mL filtered	6.8 × 10^4^ ± 1.6 × 10^4^	4.8 × 10^4^	9.3 × 10^4^
Min. CFU/mL filtered	5.8 × 10^4^ ± 2.2 × 10^4^	1.6 × 10^4^	9.3 × 10^4^
Max. CFU/mL filtered	7.7 × 10^4^ ± 1.7 × 10^4^	5 × 10^4^	10.6 × 10^4^
% of clearance	45.1 ± 10.7	32.0	62.3
Observed clearance rate (mL/h)	18.0 ± 4.0	12.8	24.9
Estimated filtration rate (mL/h)	76.0 ± 19.0	56.0	116.0

Values resulting from the filtration analysis. *N* sample size, *Min.* minimum, *Max.* maximum, *L* shell length, *W* weight, *CFU* colony forming unit.

### *E. coli* persistence in mussels

**Figure 2:**
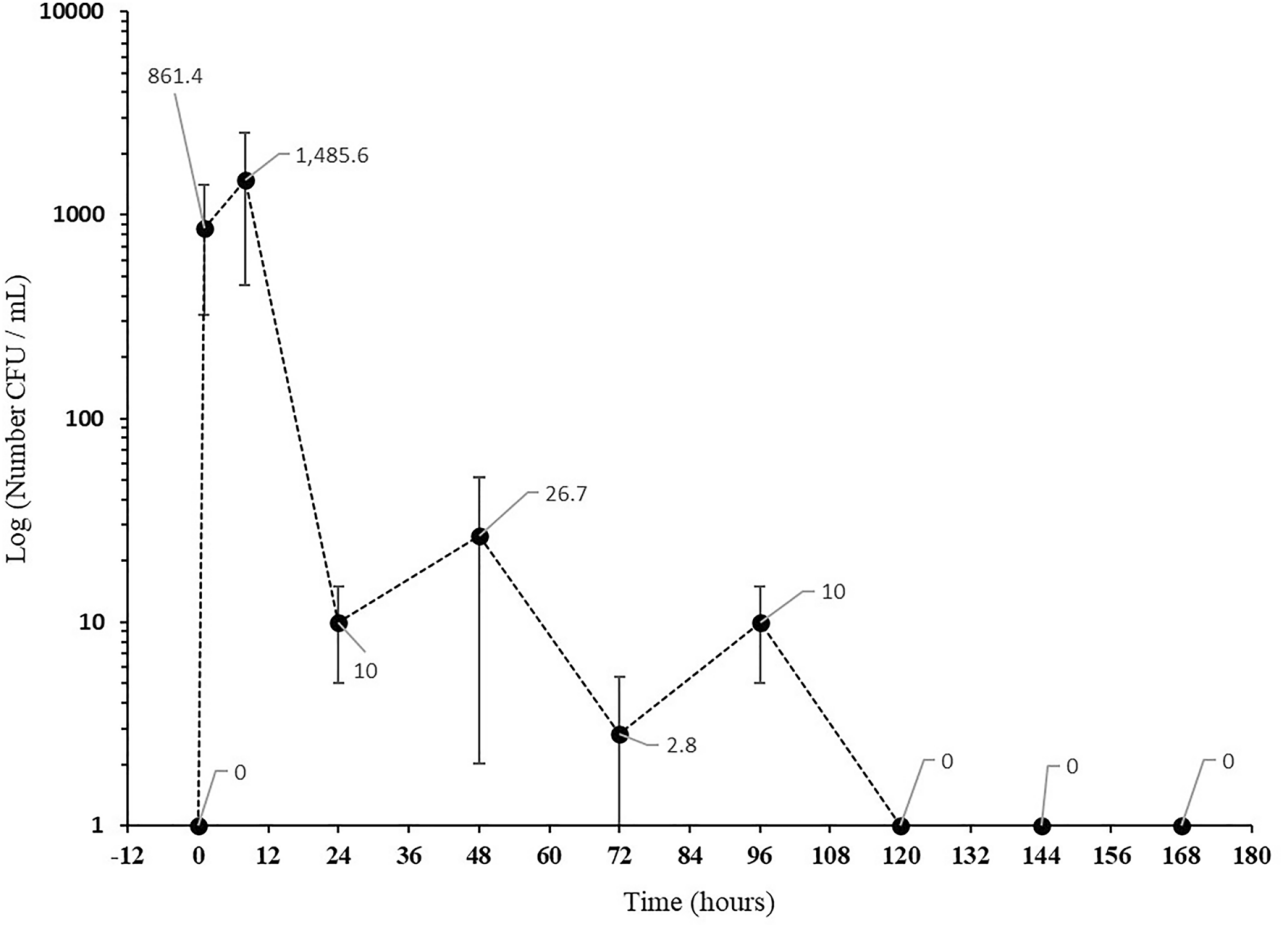
*Escherichia coli* persistence in mussels. The relationship between the average CFU/mL observed (logarithmic scale) in the soft tissue and the time (hours) in the clearance analysis phase is plotted. The labels indicate the average number of CFU/mL observed and the error bars indicate the Standard Deviation. The number of samples analysed for each time was 2 (T0), 7 (T1), 8 (T8), 3 (T24) and thereafter 3 samples.

The depuration phase ended 168 h (7 days) post-exposure (Fig. 2). Analyses of the control mussels (T0), water and microalgae feed showed that they were negative for the presence of *E. coli*. A high concentration of *E. coli* was observed in the soft tissue of the specimens at the first two sampling time points, drastically declining to a few units per mL after the first 24 h. The presence of *E. coli* was detected in the tissue up to 96 h (4 days) after exposure, and in the culture water, up to 48 h but not later than 96 h after exposure.

## Discussion

Overall, the analysed specimens of *U. mancus* showed low biometric variability. The linear equations obtained may prove informative for allometric and filtration rate studies of *U. mancus* within a size range of 17–34 mm. We observed a strong correlation between the length and the weight of their parts. In addition, it was possible to verify that the uptake of *E. coli* by the mussels was carried out successfully. Usually, both, clearance and filtration rates increased with size^36,38–41^. In our case, the correlation between size and the observed clearance and filtration ratio was not significant. This result may be due to the sum of factors such as exposure time and insufficient adaptation, the stress caused to individuals and the condition index (relationship between shell length and soft body weight). It is known that the variability of the condition index influences allometric relationships associated with clearance and filtration rate^42^. Some authors recommend the use of dry instead of wet weight to estimate clearance and filtration rates as tissue weight is known to fluctuate due to changes in season or health status^35^. The clearance ratio was analysed to discriminate the possible effect of size; however, no statistically significant effect of size on clearance ratio was observed in our data. The soft tissues analysed were used for the microbiological assays and their dry weight could not be directly measured. Therefore, an approximation of dry weight was used to estimate clearance and filtration rates. In our study, the filtration rate appeared to be much higher than the clearance rate. A possible explanation is that the mussels recirculate a greater quantity of water without retaining all of the particles in suspension as they must select and manage them internally. In the preliminary clearance experiment, pseudo-faeces, due to overfeeding, were observed, which may also explain the difference between filtration capacity and clearance.

Previous studies with *D. polymorpha* have shown that the reduction of pathogens from water was significant between 4 and 24 h post-exposure and pathogen-specific, for instance, poliovirus titre, after 24 h; rotavirus titre, after 4 h; and *E. coli* counts, about 1.5 log after 4 h and complete, after 24 h^25^. Our experimental analysis demonstrated that *U. mancus* contained the highest concentration of viable *E. coli* after one hour of exposure, which drastically declined 24 h after exposure. However, low numbers of CFUs of *E. coli* were detected up to 96 h post-exposure. Thus, *U. mancus* has the capacity to retain viable pathogen for up to 4 days. Additionally, *E. coli* was only detected in the water over a short period of time. Possibly, filtered *E. coli* are ingested and digested, and its amino acids incorporated into the bivalve proteins, as previously observed with *D. polymorpha*^43^.

Little is known about the long-term survival of pathogens inside bivalves or the impact of their presence on the health status of the host. Previous studies have reported that *Aeromonas* spp. can be lethal for *D. polymorpha*^18,25,44^. We showed that *E. coli* is not toxic for *U. mancus* at a dose of 1.5 × 10^5^ CFU/mL under laboratory conditions, and it can be retained for a maximum of four days. These results are important to understand host-bacterial interactions and to protect endangered aquatic species from potential pathogens resulting from human activities or the degradation of the ecosystem.

Organisms that detect environmental risks or changes in an ecosystem are considered sentinels^9,10^. In this sense, marine and freshwater bivalves should generally be considered sentinel species due to their filtration feeding system, which confers them with the capacity to concentrate organic (bacteria, phytoplankton, organic fragments) and inorganic particles from water^25,34,38^. For example, the *E. coli* removal capacity of oysters and hard-shelled clams^45^, common marine mussels^46^ and freshwater bivalves such as *D. polymorpha*, *Anodonta californiensis*, *Corbicula fluminea* and *Caruncilina texasensis*^14,15,19,25,43^ has been broadly assessed. Together, these studies show that clearance ratios and size limits are highly variable and species-specific and, moreover, both zoonotic bacteria and parasites such as *Aeromonas* spp., *Flavobacterium* spp., *Pseudomonas fluorescens*, *Shewanella putrefaciens*, *Shigella* spp., *Cryptosporidium parvum*, *Giardia duodenalis* and *Toxoplasma gondii* have already been detected in the soft tissue of several freshwater mussels^23–25,46^, further supporting a sentinel role for mussels.

In summary, we have demonstrated for the first time, the capacity of the freshwater mussel *U. mancus* to clear and accumulate viable *E. coli*, confirming their significant functionality in ecosystem service by clearing potential pathogens in freshwater ecosystems. Additionally, we have established basic knowledge to develop non-invasive molecular techniques enabling the use of *U. mancus* as a sentinel to study the presence of Enterobacterales in freshwater environments. Data generated from this study intends to enhance the value of this endanger species to raise authorities’ awareness to invest in its conservation and strengthen the One Health strategy.

## Methods

### Mussel selection

In April 2019, 38 juveniles (each around 3 years old) of *U. mancus* were collected from the freshwater mussel breeding center at Banyoles Lake (Consorci de l’Estany, Girona, NE Spain). An additional five individuals were collected for preliminary clearance tests (see below). The specimens and their shells were cleaned with sterile water prior to placement in a 100-L aquarium without sediment, kept without artificial lighting and with gentle aeration. The water used to fill the aquarium (hereafter referred to as filtered lake water) was taken from the oligotrophic Banyoles Lake, filtered at 100 microns and disinfected under ultraviolet light before use (used the UV sterilizer Aqua Medic Helix Max 2.0 [18 W] and a aquarium pump with throughflow rate 500 l/h). The mussels were first subjected to a fasting process (cleaning phase) for 48 h to ensure that they had purged any and expelled pseudo-faeces and faeces prior to the experiment.

### Inoculum preparation

For the inoculum, an *E. coli* isolate with a known mechanism of resistance to cephalosporins was selected (*E. coli bla*_*CTX*-M-14_). The isolate, obtained from the cryobank (Identification code: E1V1C17a) at the IRTA-CReSA Research Center (Bellaterra, Spain), was cultured on MacConkey agar medium supplemented with ceftriaxone (2 mg/L) at 37 °C for 24 h. A conservative final working concentration of *E. coli* equal to 1.5 × 10^5^ CFU/mL was prepared to ensure non-toxicity to the mussels. This concentration was chosen on the basis of two previous studies performed with *Dreissena polymorpha* were incubated with a 10^5^ CFU/mL inoculum of opportunistic bacteria and 10^7^ CFU/mL of an *Aeromonas* sp. inoculum^18,44^. The *E. coli* inoculum used here was prepared at an initial concentration of 1.5 × 10^8^ CFU/mL (turbidity of a 0.5 McFarland standard) and applied to each of the samples during the exposure phase.

### Preliminary clearance test

According to the models proposed by Kryger and Riisgård^36^ and Ostrovsky et al.^37^, unionid mussels of the specimen sizes used in this study filter approximately 50 mL of water per hour. As a preliminary test, we evaluated the capacity of five *U. mancus* mussels to filtrate total water content under laboratory conditions. Each *U. mancus* was placed into a 50 mL Falcon™ tube with water inoculated with a microalgae suspension (lyophilized phytoplankton, Easy reefs^®^, Fitoplancton Marino SL, Spain). The excretion of pseudo-faeces containing phytoplankton through the mantle borders, due to overfeeding, was observed in all five individuals and considered as proof that the water had been filtered. These individuals were not used again in subsequent experiments.

### Exposure phase

Thirty-six mussels were exposed to *E. coli* bacteria for one hour at 18 °C under laboratory conditions. Two naiads served as negative controls: they were kept in only filtered lake water and were not exposed to the bacteria. A filtered lake water sample was also collected as a control. For the exposure phase, 50 mL Falcon™ tubes were filled with 40 mL of filtered lake water. Each mussel was placed in a Falcon™ tube in a natural position, with the posterior extremity facing upwards, to facilitate the filtering process. Once all specimens opened, and their siphons were visible (about 10 min after placement in the tubes), a micropipette was used to inoculate the water with 40 µL of the 1.5 × 10^5^ CFU/mL *E. coli* solution. At the end of the exposure time (1 h), the exposed mussels were removed from the tubes using long sterile tweezers, placed in a 500-mL beaker and rinsed five times with filtered lake water to minimize the transfer of *E. coli* to the depuration tank. In addition, water samples were collected from nine of the Falcon™ tubes at the end of the exposure phase to estimate the clearance rate, i.e. the proportion of bacteria removed by the mussels during the exposure time in relation to mussel size.

### Depuration phase and sampling

The treated mussels were maintained in the depuration tank in a laboratory room kept at a constant temperature of 18 °C to avoid thermal stress. We used a culture system that we had previously established to successfully maintain mussel survival for at least 26 days in the laboratory. It consisted of a plastic tank (a 25-L Tupperware container) filled with 16 L of filtered lake water in which a semi-floating PVC cylinder, 25 cm in diameter, was installed. The cylinder had a 500-µm mesh as a bottom. A 1-cm layer of sterilized silica sand (grain size: 0.5–1 mm) was placed on top of the mesh bottom. A submersible water pump (Syncra Nano Multifunction Pump, SICCE^®^) was attached to the tank wall for uniform mixing, constant recirculation and aeration of the water. Water temperature was monitored using a submersible thermometer. The mussels were examined and fed a microalgae suspension (lyophilized phytoplankton, Easy reefs^®^, Fitoplancton Marino, SL) daily. Feed samples were also analysed to exclude the presence of *E. coli*.

Mussels were analysed for the presence of the inoculum one hour (T1), 8 h (T8) and thereafter, every 24 h (T24, T48, etc.). A total of three mussels were collected each sampling time point. Mussels of different sizes were selected at each time point to test for the possible effect of size on clearance rate and, consequently, bacterial removal. One litre of water was sampled from the tank at T48, T96 and T168, and tested for the presence of *E. coli*. Every 24 h, the tank was emptied, washed and disinfected with a 2% sodium hypochlorite solution, and the sand was rinsed and autoclaved. The remaining mussels were transferred to a new tank.

Sampled mussels were individually weighed and measured prior to their dissection. The entire soft body was homogenized using a vortex in 1 mL of sterile phosphate-buffered saline solution (PBS composition: Sodium Chloride [1.37 M], Potassium Chloride [0.027 M], Phosphate Dibasic [0.1 M]; Potassium Phosphate Monobasic [0.018 M]) at pH = 7.2. From each of the resulting suspensions, 100 µL were spread, using a Drigalski spatula, onto plates containing two different media: MacConkey agar and MacConkey agar supplemented with ceftriaxone (2 mg/L). Duplicates were made for all cultures. Plates were incubated at 37 °C for 24 h, and colonies were manually counted. MacConkey agar medium was used as an indicator of Enterobacterales colony growth in general as a reference plate. MacConkey agar medium supplemented with ceftriaxone as a selective medium for the added strain. Verification method that provides comparable information between the diversity of non-selected colonies and the marked *E. coli* strain.

Water samples were filtered with a paper filter (Durapore^®^ membrane filters, 0.45 µm). Filters were then individually homogenized in 10 mL of PBS, and 100 µL of each homogenate were cultured on MacConkey agar and MacConkey agar plus ceftriaxone plates and incubated at 37 °C for 24 h. The growth of *Escherichia coli* from the plates was assessed without counting the number of colonies. The result was indicated as positive if colonies are observed and negative no colonies.

### Statistical analyses

The relationship between the different biometrical parameters (total weight and shell, soft body, free water and dry soft body weights) and the total individual length was analyzed by a linear regression model. A coefficient of determination (*R*^2^) and a linear equation (slope and intercept) were obtained for each relationship. Clearance rates were analysed by Spearman correlation. All analyses were performed using IBM SPSS Statistics 22. Basic descriptive statistics including mean, standard deviation, minimum and maximum values were calculated using Microsoft Excel.

### Approval for animal experiments

The experimental use of endangered captive-bred *U. mancus* was authorized by the Subdirecció General de Biodiversitat i Medi Natural del Departament de Territori i Sostenibilitat from Generalitat de Catalunya (Code: SF/0507/2019). All applicable institutional and/or national guidelines for the care and use of animals were followed.

## Data Availability

All data generated or analysed during this study are included in this published article.
